# A Snapshot of the Response from UK-based Clinical Trials of Investigational Medicinal Products to COVID-19

**DOI:** 10.7759/cureus.10613

**Published:** 2020-09-23

**Authors:** Sandra Neumann, Emily Henderson

**Affiliations:** 1 Neurology, Bristol Medical School, University of Bristol, Bristol, GBR; 2 Internal Medicine: Geriatrics, Royal United Hospitals Bath National Health Service Foundation Trust, Bath, GBR

**Keywords:** ctimp, response to covid-19, conduct, older adults, ageing, neurology, methodology, survey, risk assessment, clinical trials

## Abstract

Background/Aims

Coronavirus Disease 2019 (COVID-19) has presented an unprecedented challenge for non-COVID related clinical trials of investigational medicinal medicines (CTIMPs). These challenges are considerable for trials run in high -risk groups, such as older adults. Clinical trials must ensure the safety of their participants, whilst also considering the potential, and often long-term, benefits of the trial intervention to public health. Here we sought to provide a brief perspective on the response and conduct of CTIMPs relevant to older adults and neurology in the UK to the COVID-19 pandemic.

Methods

We performed a cross-sectional study, surveying CTIMP teams running trials broadly relevant to older adults and neurology in the UK, as well as sponsors and Clinical Trials Units (CTU), to understand the response and preparedness to the pandemic.

Results

Due to the pandemic, active recruitment has been suspended in more than half of the trials. The primary driver for the temporary halt of recruitment activity was considerations of patient safety. Interestingly, the majority of trials, sponsors and CTUs did not consider pandemic or epidemic outbreaks in their risk assessments before January 2020.

Conclusion

These findings support the need to re-evaluate the risk-management approach whereby clinical trials establish contingency plans for predicted but rare events to minimise the disruption to recruitment and clinical trial delivery.

## Introduction

Clinical trials have faced an unprecedented challenge in the light of COVID-19. On March 11, 2020, the World Health Organisation (WHO) declared a pandemic which unequivocally complicated the conduct of clinical trials. Importantly, urgent strategies were required to ensure the safety of enrolled participants, weighing up the potential risks to participants of acquiring COVID-19 versus the often long-term, benefits of the trial intervention to public health [1].

In the United Kingdom, the Health Research Authorities (HRA) published a response to the pandemic advising clinical trials to evaluate the risk-benefit of their trial in the broader context of the impact of COVID-19 on National Health Service (NHS) staffing, restriction of movement and government advice [2]. The National Institute for Health Research (NIHR) strategic response stated that all non-COVID-19 related trial set-up activity would be temporarily suspended, and trials in the recruitment stage halted on a case-by-case basis to ensure the prioritisation of COVID-19 studies and to enable the redeployment of clinical staff to frontline care [3]. Since then, the Medicines and Healthcare products Regulatory Agency (MHRA) have issued pragmatic guidance on how to manage clinical trials during the pandemic, including practical guidance on the safe delivery of Investigational Medical Products (IMPs) to participants in self-isolation, remote monitoring of trials, protocol deviations and ensuring timely and appropriate measures for pharmacovigilance [4].

Guidance issued by the major UK non-commercial funding bodies stated that funding for trials in progress would not be affected by the temporary halt of the trial due to COVID-19, in the short term, and/or that the continuation of funding and no-cost extensions will be assessed on a case-by-case basis [5-7]. We recognised that responses and interpretation of these directives varied on both a trial and institutional level. Therefore, we sought to describe and understand the decisions and action in response to COVID-19 for clinical trials of investigational medicinal products (CTIMPs) that were relevant to older adults and those with neurological conditions. These groups were chosen as a subset of those classed by the UK government as 'clinically vulnerable people' who were advised to take particular care to minimise contact with others outside their household [8].

This study aimed to determine the response to the COVID-19 pandemic at; a) clinical trial management level and, b) an institutional level. We sought to determine the degree to which the pandemic impacted trial activities. As well, we set out to understand the significant drivers behind decisions affecting the running of CTIMP trials and the pandemic preparedness in terms of pre-existing risk assessments.

## Materials and methods

This is a retrospective, cross-sectional survey of clinical trials taking place in the UK. Ethics approval was received from the University of Bristol on the 5th May 2020 (reference no- 104302).

The European Union Drug Regulating Authorities Clinical Trials (EudraCT) Clinical Trial Register database was searched on May 8, 2020 using advanced search terms defined as studies in the UK (Country), Elderly (Age range), Ongoing, Prematurely ended, Restarted or Temporarily halted (Trial status) and Phase I, II and II (Trial phase). Gender selection was not specified. The study included trials that opened between March 1, 2015, and March 16, 2020. The search yielded a total of 2034 trials.

The results were screened based on the patient population for conditions primarily affecting or relevant to older adults or neurology. The following conditions and studies were excluded: alcohol and substance abuse-induced disorders of the liver and lungs, anaesthesia, asthma, cystic fibrosis, dermatology, endocrinology including type 2 diabetes, gastrointestinal and metabolic disorders, haematology, infectious diseases, ITU admission, oncology, post-surgical interventions, psychiatric disorders that were not secondary to a known neurological condition, rare genetic disorders, rheumatology, trauma and transplant studies.

A total of 265 CTIMPs met the inclusion criteria and were emailed an invite to take part in the study. Emails were obtained from publicly available sources, including trial registries and websites. The person responding on behalf of the CTIMP completed electronic consent before participating. CTIMPs were surveyed on their response to COVID-19 concerning recruitment, IMP management and follow-up activity. We explicitly enquired as to whether the CTIMP risk assessment referenced pandemic management before the pandemic (i.e. before January 2020).

The sponsor organisations of the 265 CTIMPs were identified via EudraCT and invited to take part using contact details via publicly available sources such as trial registries. This survey included a total of 149 sponsor invitations (given that some sponsors provide support to more than one study). Also, 46 clinical trial units (CTUs) who are fully registered with the UK Clinical Trials Research Network (UKCTRN) were identified via the UKCTRN website. Of these, seven CTUs specialised in areas beyond the scope of the trial criterion (e.g. paediatrics or cancer research) and were excluded. A total of 39 CTUs were invited to take part in the survey.

Sponsors and CTUs were surveyed on the advice provided to trials concerning recruitment during the COVID-19 pandemic as well as preparedness in terms of crisis management plans or risk assessment considering the effect of a pandemic on trial activity. We also collected data on the number of CTIMPs supported by the sponsors and CTUs, excluding COVID-19.

The results are presented as percentages and descriptive statistics.

## Results

**Figure 1:**
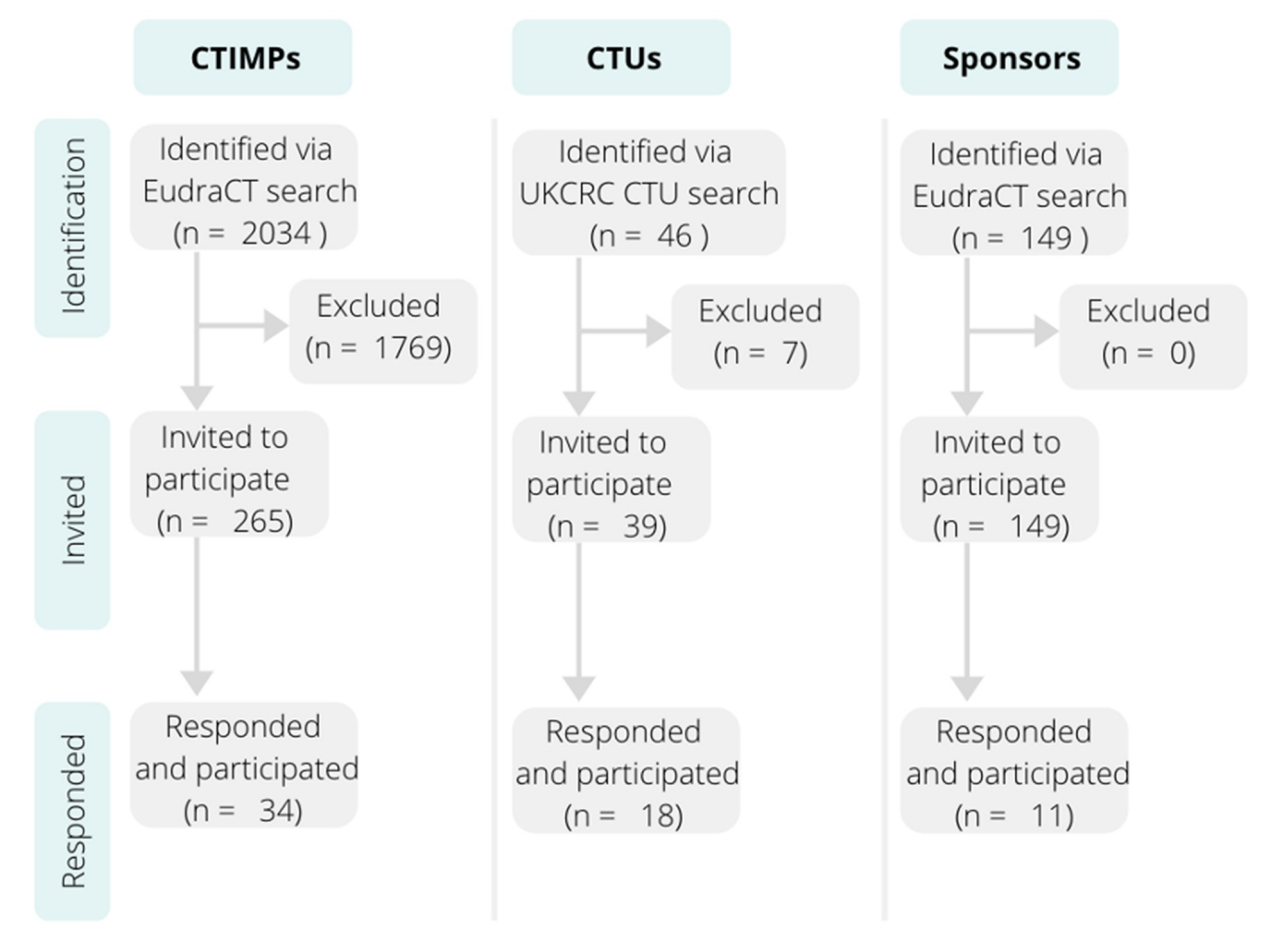
Consort diagram of the approached trials, clinical trials units and sponsors. CTIMPs- clinical trials of investigational medicinal medicines; CTUs- Clinical Trials Units; UKCRC CTU- UK Clinical Research Collaboration Clinical Trials Units; EudraCT- European Union Drug Regulating Authorities Clinical Trials

The highest response rate was received from CTUs (46% response rate), compared to the response rates from trials (12% response rate) and sponsors (7% response rate) which were markedly lower. This is illustrated in Figure 1.

Trial characteristics

Of the 265 invited CTIMPs, 34 trials took part in the survey. The majority of the responses pertained to trials in the fields of Neurology or Psychiatry (44%) followed by Cardiology (41%) with the remaining trials falling under respiratory, ophthalmology or trauma in older adults. 5 trials were single-centre trials, and 29 trials were multicentre. 14 trials were international with sites in the UK, whilst 20 were UK based.

On the 16th of March 2020, when the UK Government announced the ‘Stay Home’ directive, of the 34 trials, 3 were in set-up, 16 trials were actively recruiting, 2 trials were suspended, 12 had completed recruitment, 1 trial was stopped permanently.

Overall, 24/34 (71%) trial teams had considered alternative ways to deliver the trial in order to reduce the risk to participants and/or healthcare staff. Of these 15/34 trials (44%) were able to implement changes to deliver the trial during COVID-19. Seven trials (21%) reported that they did consider this but did not implement the changes because the institution, funder or other organisation such as the NHS Trust had requested the suspension of the trial. Alternative methods of delivery were considered by 2 trial teams (6%) who were unable to implement these changes and in one case this was not considered to be applicable to the trial. Nine trial teams (26%) suggested that they had not considered alternative ways of delivering the trials.

Impact of COVID-19 on recruitment

**Figure 2:**
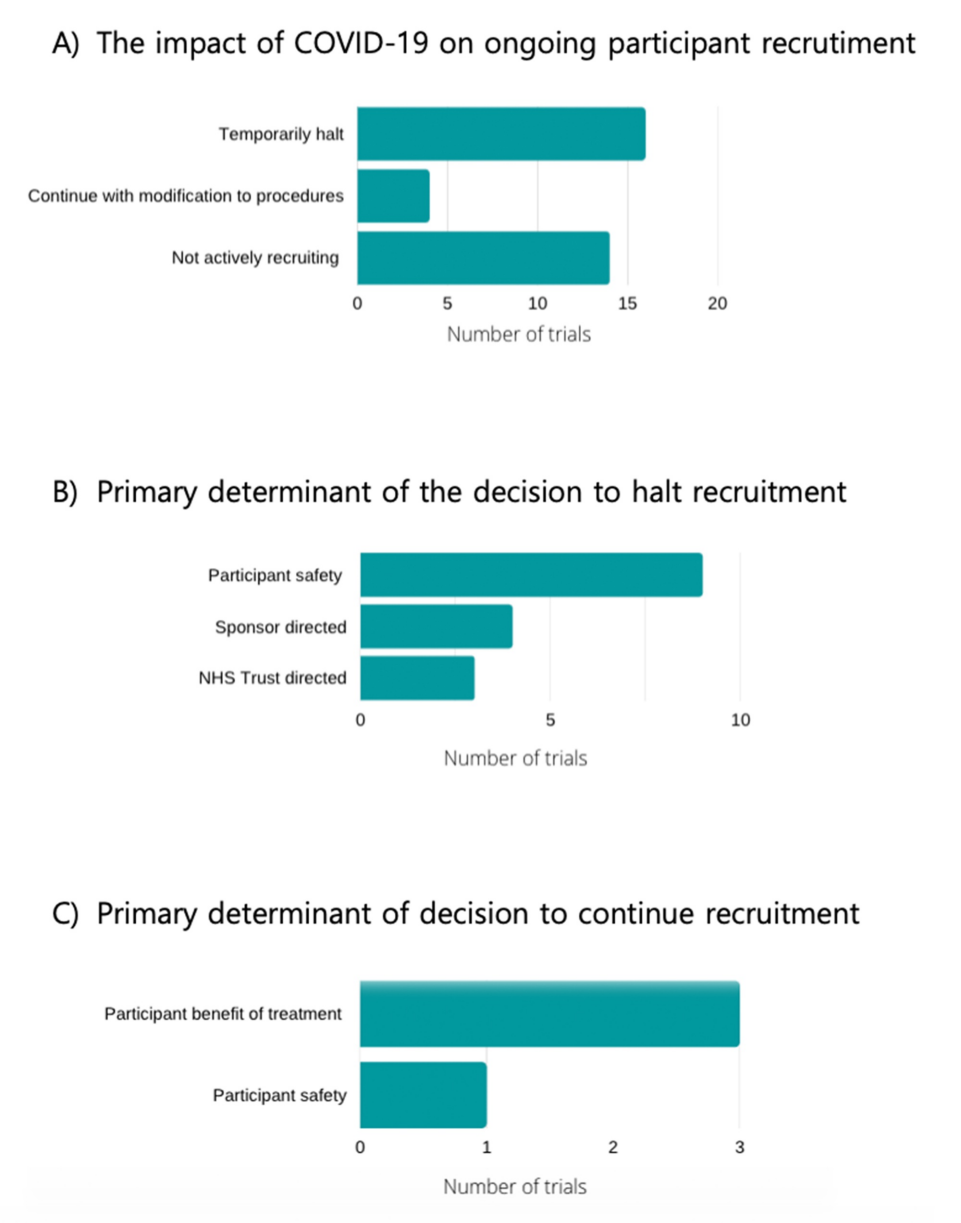
Trial decisions on recruitment during COVID-19 The first panel (A) shows the distribution of trials that continued or halted recruitment. The second panel (B) shows the primary factor for the decision to halt recruitment during COVID-19 and the third panel (C) shows the primary factor in the decision to continue recruitment during the pandemic. NHS- National Health Service

Sixteen out of 34 trials (47%) temporarily halted recruitment during the pandemic, 4/34 trials (12%) continued recruitment with modification to procedures, and the remaining 14 trials (41%) were not actively recruiting e.g. studies in the follow -up phase. Data on recruitment decisions as well as the primary factor for the decision is shown in Figure 2. Of the trials that halted recruitment, the majority of 9/16 (56%) identified participant safety as the most important factor in the decision. Seven trials halted recruitment as directed by the sponsor (4/16 (25%)) or by an NHS Trust (n=3, (19%)). The decision halt recruitment was driven by several factors. Patient safety and staff considerations that included e.g. healthcare staff redeployment were independent factors in 11/16 trials (69%). Institutional guidance from the sponsor influenced decision making in 8/16 trials (50%), and an NHS Trust directive influenced 7/16 trials (44%). Guidance from funders had less bearing influencing the decision in only 2/16 (5%) trials. Furthermore, logistics such as drug delivery was a factor in decision making in only 5/16 trials (31%). Other factors that were considered included the risks associated with treatment, data integrity/validity considerations and participant benefit of treatment.

Of the four trials which continued recruitment during COVID-19, the primary driver in 3/4 (75%) of trials was potential participant benefit of treatment and participant safety 1/4 (25%). Concerning the factors influencing the decision to continue recruitment with modified procedures, a single trial identified participant benefit of the treatment, and one identified the risk associated with treatment withdrawal as influencing the decision. In 3/4 (75%) trials, funding was a factor. 

Impact on the Investigational Medicinal Product intervention

**Figure 3:**
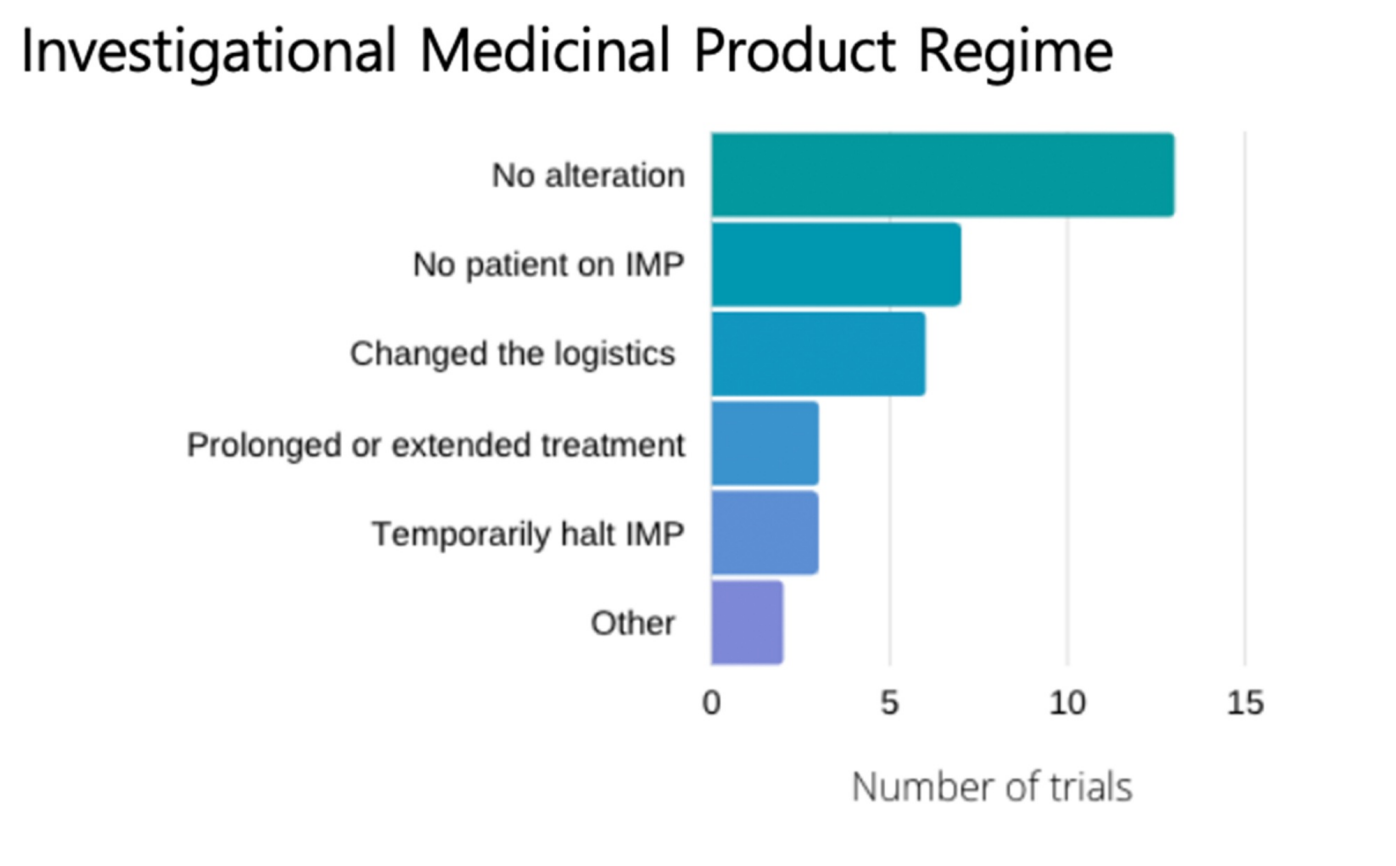
Adjustments to the regime or protocol for the investigational medicinal product. IMP- Investigational Medical Products

The impact of COVID-19 on IMP regimes is shown in Figure 3. For participants enrolled in trials, 13/34 (38%) made no changes to the IMP regime. 3/34 (9%) temporarily discontinued the IMP, and 3/34 trials (9%) reported that they prolonged or extended the treatment duration. 7/34 trials (21%) were not administrating the IMP at the time. 6/34 trials (18%) changed the logistics of the IMP management to allow participants to be titrated, infused or receive the IMP via post as to avoid the need to attend face-to-face appointments or collections.

Free-text feedback highlighted that the decision regarding the IMP regime continuation or alteration was driven by clinical and/or practical considerations for the participants.

Follow up assessments

**Figure 4:**
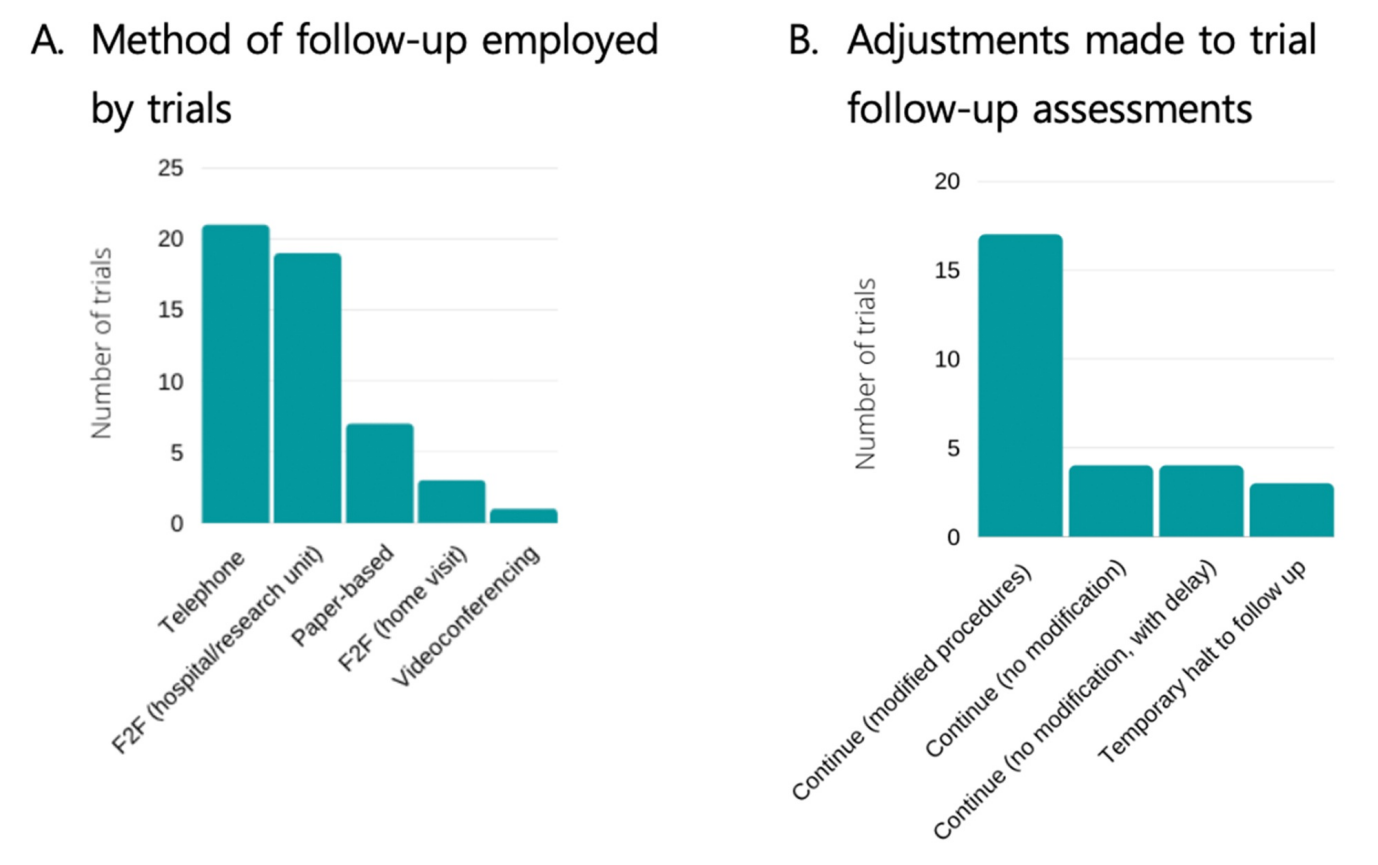
Follow up assessments. Follow up assessments reported by trials (A). Note some trials may have more than one type of assessment. The trials adjustment of follow up activity in response to COVID-19 (B). F2F: Face-to-face

Data on follow up assessments are shown in Figure 4. Thirty-two trials had follow-up assessments. Of the 32 trials, 21 trials (66%) had telephone follow-ups. 19/32 trials (59%) had face-to-face follow-ups at a hospital, clinical or research centre. Three trials (9%) had face-to-face visits at home, and 7/32 trials (22%) were paper-based assessment (e.g. questionnaires). One trial reported using video-conferencing for follow-up assessments. Half of the trials reported that they were planning to continue to follow up activity with modified procedures (17/32 trials, 53%), whilst 4/32 trials (13%) planned to continue to follow up as planned and 4/32 trials (13%) planned to continue to follow up as without modification to the procedure, but with delayed timelines. Three trials (9%) reported a temporary halt to the follow-up activity. The remaining trials had no change to the follow-up activity either because the trial was in set up or the follow up had been completed.

Trial Risk Assessment

**Figure 5:**
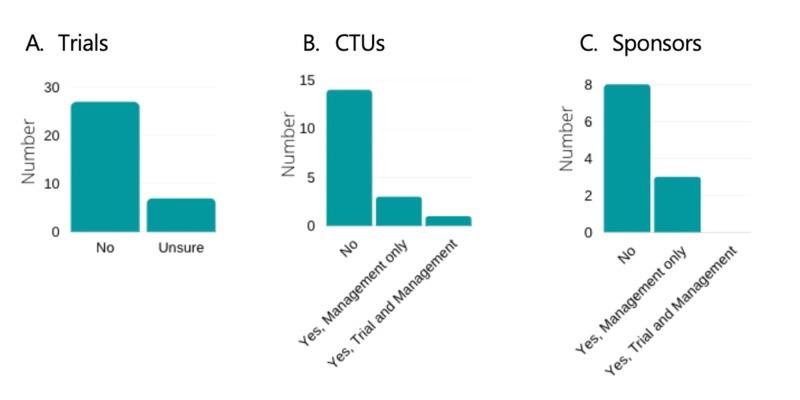
Risk assessments. Risk assessments including reference to  ‘pandemic’ and/or ‘epidemic’ for Trials (A), CTUs (B), and Sponsors (C), respectively. CTU- Clinical Trial Units

None of the trials surveyed identified 'epidemic', 'pandemic' or other 'public health emergency' as listed on the trial risk assessment. Twenty-seven trials (79%) stated that their risk assessment did not list these terms, whilst 7/34 (21%) trials were unsure. This data is shown in Figure 5.

CTU response

Eighteen CTUs responded to the survey and were supporting 17 (range 0 and 14 per unit) actively recruiting CTIMPs on March 16 2020. Four CTUs had CTIMPs that remain actively recruiting (excluding COVID-19 studies), with 3 CTIMPs being relevant to older adults.

**Figure 6:**
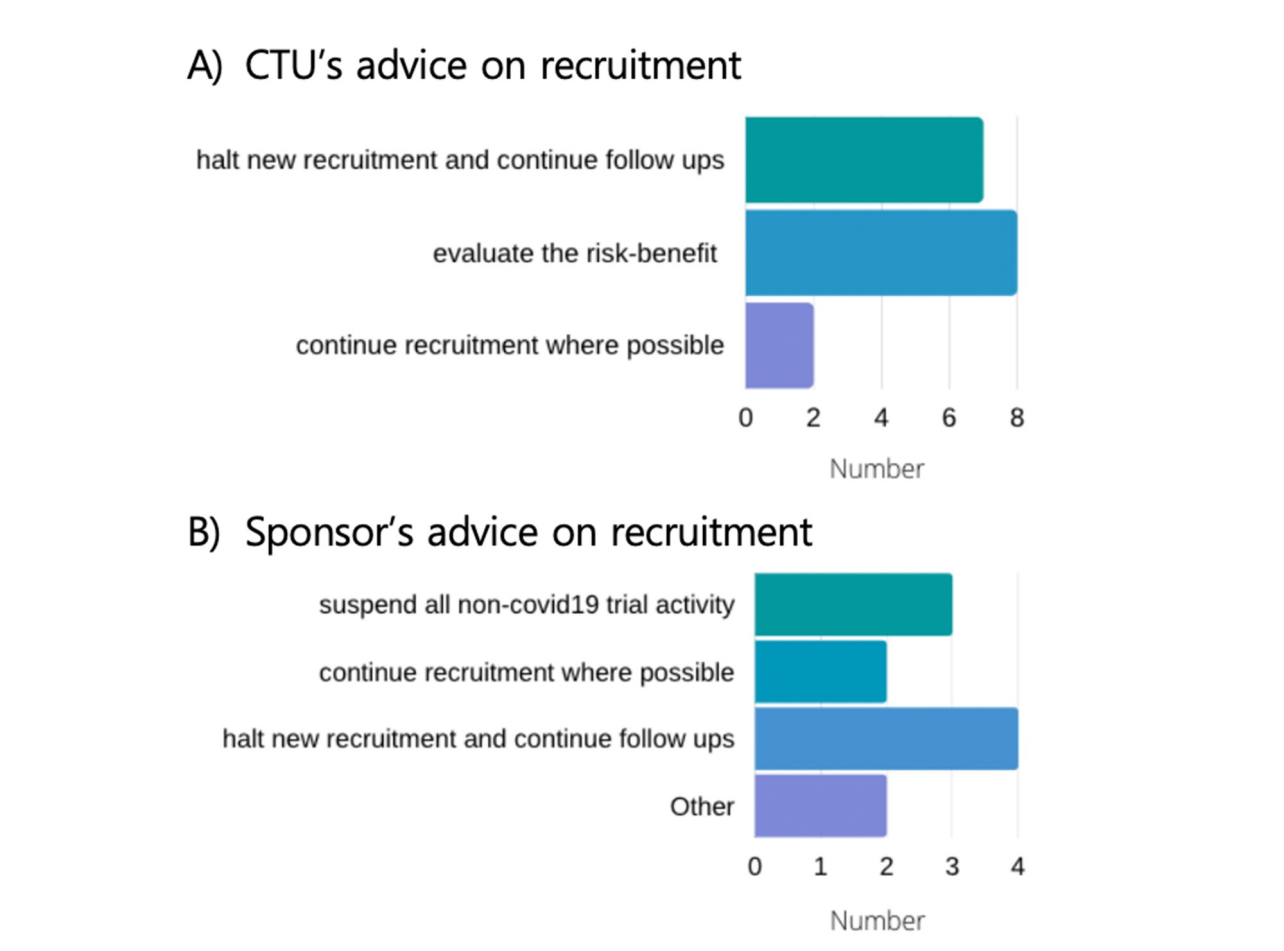
Advice on recruitment. Advice on recruitment to trial teams in response to COVID-19 by CTUs (top panel (A), n=18) and Sponsors (lower panel (B), n=11). CTU- Clinical Trial Units

CTU level guidance in response to the COVID-19 crisis advocated halting new recruitment and continue to follow-ups in 7/18 units (39%). Eight CTUs (44%) indicated that the trial team (trial managers or chief investigators) should evaluate the risk-benefit of their trial with regards to continuing or halting recruitment. Two units (11%) encouraged continued recruitment where possible. These data are illustrated in Figure 6.

Prior to January 2020, 4/18 (22%) CTUs had an action plan or a crisis management plan in place describing the steps to be taken in the case of a pandemic (or epidemic), 4/18 (22%) CTUs reported non-specific contingency plans or guidance was in place for unexpected events but not specific to a pandemic (or epidemic), and 8/18 (44%) CTUs had no pandemic (or epidemic) management plan.

One CTU reported pandemic or epidemic prior to January 2020 mentioned in their risk assessment at both trial and management level. Three CTUs (17%) reported mention of pandemic or epidemic in their risk assessment prior to January 2020 but only at the management level. The remaining 14/18 (78%) CTUs did not mention pandemic or epidemic in their risk assessment prior to January 2020. This data is illustrated in Figure 5.

Preparedness of Sponsors

Eleven sponsors replied to the survey. Of these, three were commercial sponsors, and 8 were non-commercial sponsors. On March 16 2020, the sponsors supported a total of 23 actively recruiting CTIMPs (range 0-4 per unit). Of these 2 CTIMPs remained actively recruiting with no suspension of activities excluding any COVID-19 studies.

Two sponsors (18%) encouraged recruitment to continue where possible, four sponsors (36%) advised to halt new recruitment but continue to follow up activity, and three sponsors (27%) advised investigators to suspend all non-COVID related trial activity. The remaining two reported that the response was either led by the NHS trust or did not give specific advice to CTIMPs as no CTIMP was open. These data are shown in Figure 6.

Prior to January 2020, 3/11 (27%) sponsors had an action plan or a crisis management plan in place describing the steps to be taken in the case of a pandemic (or epidemic). One sponsor reported non-specific contingency plans were in place, but not specific to a pandemic. Seven sponsors (64%) reported that they had no pandemic (or epidemic) management plan in place prior to January 2020.

Three sponsors (27%) reported pandemic or epidemic prior to January 2020 mentioned in their risk assessment at but only at management level. None of the sponsors reported mention of pandemic or epidemic prior to January 2020 in the risk assessment at both trial and management level (0%). The remaining eight sponsors (73%) did not mention pandemic or epidemic in their risk assessment prior to January 2020. This data is shown in Figure 5.

## Discussion

During the pandemic, there was a rapid shift in focus and prioritisation to research that tackled COVID-19 [2-4]. The redeployment of resources and expertise led to activity in CTIMPs in other 'non-COVID' disease areas being suspended [3]. 

To further understand and quantify this broader impact, we surveyed clinical trial teams and sought data from supporting CTUs and sponsors. We identified >2000 potentially eligible trials. The response rates from clinical trials and sponsors were low and we anticipate that this may have been a different function of the pandemic. The majority of those that responded paused recruitment during COVID-19, but follow-up activity and IMP regimes continued mostly unchanged. This suggests that whilst recruitment activity was heavily affected, trials were mostly able to proceed or accommodate changes which allowed them to continue the trial activity for those participants enrolled before the COVID-19 pandemic. Such changes included modification to the follow -up procedures, adjusting time scales for follow-ups and in some cases changing the logistics of the IMP to allow for home delivery to participants. Participant safety or potential benefit was the primary driver that determined the course of action taken during the pandemic.

Very few institutions (CTUs and sponsors) had considered the pandemic impact on trials before January 2020. At a trial level, no risk assessment mentioned pandemic or epidemic, suggesting little preparatory activity to mitigate the risk was undertaken before the outbreak.

The UK National Risk Register of Civil Emergencies 2017 [9] documented an increased likelihood of emerging infectious diseases relative to the year 2015. However, the impact severity (ranked 3 out of 5) and likelihood of occurrence (ranked 4 out of 5) in the five years following the report's publication was considered equivalent to low air quality, heat waves and space weather. Nonetheless, the government continued to acknowledge the high impact severity (5 out of 5) of a potential flu pandemic. However, the likelihood was considered equivalent to that of other emerging infectious diseases (4 out of 5). Despite the relatively high likelihood of a (flu or emerging infectious disease) pandemic on the national risk register, this risk was primarily omitted in risk assessments before January 2020 at both trial and institutional level. Perhaps unsurprisingly, little guidance exists as to where the responsibility falls for assessing and adapting risk mitigation protocols for national and/or systemic risks not specific to the trial environment, such as the emergence of a pandemic.

As such, the preparedness of trials to respond and adapt to an epidemic, pandemic or local outbreak of infectious diseases which increases the risk to the vulnerable populations appears, in retrospect, to be a worthwhile exercise. For example, most trials were able to adapt follow up procedures to allow some trial activity to continue whilst recruitment was suspended in more than half of the trials actively recruiting prior to the pandemic.

Strengths and limitations

The study provides insight which may be helpful to the future planning and preparedness of trials not only to a potential second wave of COVID-19 but also to other local and national infectious disease outbreaks. The survey focused on the key aspects of trial activity for CTIMPs; namely, recruitment, follow up and IMP management. This allowed us to assess specifically the type of trial activity impacted by the novel health emergency. The study was conducted in a subgroup of people who are listed as 'high clinical risk' by UK government officials. Therefore, the CTIMPs surveyed would encompass those who are most likely to be impacted by the pandemic.

The low response rate from trials and sponsors is such that we would seek to see the findings reproduced and considered in other populations and disease areas before definitive conclusions could inform future policy. Whilst we purposefully, but pragmatically, limited the search criteria to include studies relevant to older adults and neurology, some trials may have been omitted which could be of relevance given the criteria search on EudraCT, e.g. studies which were relevant to older adults but did not tag 'Elderly' on the database.

The survey was conducted in May 2020, and therefore it was issued more than one month after the UK government's 'Stay Home' directive [8]. Whilst the timing of the survey would have allowed trials to respond to the COVID-19 crisis, it may have fallen at a time where trial managers, chief investigators and sponsors were busy preparing trials to restart activity in light of the gradual easing of restrictions nationwide. The survey did not explore the process or timeline of the response to the pandemic. Some CTUs, sponsors and trials may have adapted and prepared in advance of the WHO declaration of SARS-nCOV2 outbreaks as a pandemic on March 11, 2020 [10] or the government restriction announced on March 16, 2020 [8]. For example, the surveys asked about the mention of a pandemic on risk assessments before January 2020 only; that is mentions which preceded the WHO declaring COVID-19 a public health emergency of international concern (PHEIC) [10].

Future impacts

COVID-19 has necessitated a realignment of focus on the immediate public health crisis facing the UK population. An indirect consequence of this is the potentially negative impact on the existing portfolio of clinical trials running in non-COVID conditions. Our work has demonstrated that rapid changes and flexibility of delivery partially mitigated the impact.

A new framework for risk-adapted approaches to clinical trials may be needed until a vaccine, or efficacious treatment is available [1,4]. Trial teams are exploring novel ways of delivering clinical trials including recruitment approaches, clinical assessments and IMP monitoring. These novel methods have emerged from an urgent need during an unprecedented time. The longer-term adaptation and implementation of such measures show promise to promote trial inclusivity for a heterogeneous population of older adults with and without chronic conditions which are both under-represented in clinical research and potentially at most significant risk of acquiring COVID-19 infection. 

## Conclusions

The majority of trials temporarily halted recruitment during the pandemic. Despite a lack or delayed recognition of the risk that a pandemic presented at all levels of trial oversight, the rapid implementation of novel delivery methods holds promise for the future. Our findings call for a re-evaluation of the risk-management approach to evaluate whether clinical trials may be able to find contingency plans in the future to allow less disruption to trial activity.
